# Pathogenic Tau Protein Species: Promising Therapeutic Targets for Ocular Neurodegenerative Diseases

**DOI:** 10.18502/jovr.v14i4.5459

**Published:** 2019-10-24

**Authors:** Mohammad Amir Mishan, Mozhgan Rezaei Kanavi, Koorosh Shahpasand, Hamid Ahmadieh

**Affiliations:** ^1^Ocular Tissue Engineering Research Center, Student Research Committee, Shahid Beheshti University of Medical Sciences, Tehran, Iran; ^2^Ocular Tissue Engineering Research Center, Shahid Beheshti University of Medical Sciences, Tehran, Iran; ^3^Department of Brain and Cognitive Sciences, Cell Science Research Center, Royan Institute for Stem Cell Biology and Technology, ACECR, Tehran, Iran; ^4^Ophthalmic Research Center, Shahid Beheshti University of Medical Sciences, Tehran, Iran

**Keywords:** Microtubule-associated Protein, Neurodegenerative Disorders, Tau, Ocular Neurons

## Abstract

Tau is a microtubule-associated protein, which is highly expressed in the central nervous system as well as ocular neurons and stabilizes microtubule structure. It is a phospho-protein being moderately phosphorylated under physiological conditions but its abnormal hyperphosphorylation or some post-phosphorylation modifications would result in a pathogenic condition, microtubule dissociation, and aggregation. The aggregates can induce neuroinflammation and trigger some pathogenic cascades, leading to neurodegeneration. Taking these together, targeting pathogenic tau employing tau immunotherapy may be a promising therapeutic strategy in fighting with cerebral and ocular neurodegenerative disorders.

##  INTRODUCTION

Tau is a microtubule (MT)-associated protein and the most common unfolded protein upon neurodegenerative disorders, which was described in 1975 by Weingarten and colleagues.^[1]^ This protein gene encoding (*MAPT*), being located at chromosome 17q21,^[2]^ is predominantly expressed in various regions of the human brain, mostly in neurons and to a lesser degree in astrocytes and oligodendrocytes.^[3,4]^Tau is an axonal protein, binds to MTs by its MT-binding domain, stabilizing microtubule structure and the cytoskeleton.^[5,6,7]^ Other functions of tau are their roles in cargo conveyance and signaling pathways.^[8,9]^ Tau aggregation is widely accepted as a pathological process in central nervous system (CNS), leading to neurodegenerative disorders such as: Alzheimer disease (AD) and post-traumatic brain injuries^[10]^. Since ocular neurodegeneration is similar to CNS neurodegenerative disorders, there are several evidences that pathogenic forms of tau are prone to aggregation and responsible for retinal degeneration in the subjects with ocular neurodegeneration, such as age-related macular degeneration (AMD) and glaucoma.^[11,12,13]^ With this in mind, we aimed to summarize scientific reports focusing on the role of tauopathy in the pathogenesis of neurodegenerative diseases, in particular in the eye, and to explain the role of targeted therapy as a promising therapeutic modality for pathogenic tau aggregations.

###  Tau Gene 

The *MAPT* gene include 16 exons, of which 11 are expressed in the human brain and 6 main isoforms of 37-46 kDa tau mRNA are produced by alternative splicing that results in production of six tau protein isoforms. Difference between the isoforms is dependent on the N-terminal region where the number of copies of a repeated motif consisting of 29-amino acids (0N, 1N, or 2N), and the C-terminus including either three (3R tau) or four (4R tau) MT-binding repeats.^[14,15,16]^ In human CNS, the longest tau isoform with 441 amino acids, 2N4R, has 80 Thr and Ser residues that can be modified by numerous kinases^[18]^ and a low proportion of hydrophobic amino acids, which renders tau a hydrophilic protein.^[17]^

Differential splicing that alters tau protein isoform expression occurs at every step of development and neuronal maturation.^[10]^ In the adult brain, all six tau isoforms are expressed, in contrast to fetal brain where the shortest tau isoform (0N3R) is expressed.^[16]^ In the cerebral cortex of healthy adults, approximately equal amounts of 3R and 4R tau isoforms are expressed.^[16]^ Additionally, it has been reported that there is 3R/4R ratio changes in the AD patient brains compared to the healthy subjects, demonstrating that the isoforms ratio is a determinant factor in tau pathogenicity and aggregation.

Regional splicing of tau mRNA has also been observed in human brain. The expression rate of 0N3R tau isoform in the cerebellum is lower than other regions in human brain and 4R tau isoforms are highly expressed in the globus pallidus.^[19,20]^

###  Tau Protein Structure

Tau protein has a flexible conformation with a low level of secondary structure^[21,22]^ and is subdivided into four domains with different biochemical properties. The N-terminal acidic domain with 1–150 amino acids includes two N-terminal inserts. Tau protein amino acids 151–243 are known as the proline-rich domain.^[6]^ The MT-binding domain of tau consists of four repeated motifs that are separated from each other by flanking regions, which altogether provide a structure by which the tau can bind to and stabilize MTs.^[22,23]^ Amino acids 370–441 are known as the C-terminal region.^[22]^

The N-terminal domain protrudes out of the MT surface, and although this domain does not bind directly to MTs, it has a role in MT assembly regulation and affects the attachment or spacing between MTs and other components in the cell.^[24]^ The N-terminal inserts affect the distribution of tau molecules in the cell; it was demonstrated that each tau isoform (0N, 1N, and 2N) has different subcellular localizations in the mouse brain.^[25]^ In addition, tau, via interacting with the membrane binding protein annexin A2, interacts with the plasma membrane by its N-terminal domain.^[26,27]^ The N-terminal domain can also bind to the C-terminus of p150 in dynactin protein, which has an essential role in the connection between dynein and cargoes.^[28]^ Moreover, tau isoforms have distinct protein interaction patterns; for instance, apolipoprotein A1 can bind to 2N tau isoforms; however, synaptophysin and β-synuclein attach to 0N tau isoforms.^[29]^

The proline-rich domain of tau has several recognition sites for attaching Src homology-3 (SH3)-containing proteins such as the Src family of protein kinases (Lck, Fgr, and Fyn), the p85α regulatory subunit of phosphatidylinositol 3-kinase (PI3K), bridging integrator 1 (Bin1), phospholipase C (PLC), γ1, PLCγ2, growth factor receptor bound protein 2, and peptidylprolyl cis/trans isomerases NIMA-interacting 1.^[30]^ Tau interactions with SH3-containing proteins play an important role in modulating the signaling functions of tau. Additionally, signaling pathways are associated with the activation of phosphatidylinositol and phosphatidylinositol bisphosphate, which collaborate with the tau proline-rich domain.^[31,32]^ Furthermore, the tau proline-rich domain acts as a DNA and RNA recognition site.^[33,34]^ This domain also has an important role in the inter MT spacing and intracellular trafficking^[35,36]^ as well as actin binding,^[37]^ highlighting an important role in neuronal cell signaling and neuronal plasticity.

###  Tau Post-translational Modifications 

Although several post-translational modifications, in forms of phosphorylation, acetylation, glycation, cleavage or truncation, prolyl-isomerization, polyamination, nitration, ubiquitination, oxidation, and sumoylation have been identified to modulate tau protein,^[38,39,40]^ the most well-known is phosphorylation in which the abnormal hyperphosphorylation is associated with tauopathy.^[41]^ Although phosphorylation is an essential tau modifier under physiological conditions,^[1]^ abnormal phosphorylation or hyperphosphorylation are other modifiers by which tau gets prone to aggregation.^[42,43]^ For instance, hyperphosphorylated tau forms the oligomers and neurofibrillary tangles (NFTs) that exert toxic effects on neuronal function and AD progression.^[44]^

Although 15–30 phosphorylation sites, that mostly corresponded to proline-directed sites in the proline-rich domain of tau were discovered in tau protein,^[45]^ increased phosphorylation, especially at the MT-binding domains, has also been demonstrated in association with increased MT dynamicity and reduced levels of neuronal excitability in the early stages of AD.^[46]^

Threonine${}^{175}$ (Thr${}^{175}$) is one of the important phosphorylation sites in tau protein that was first identified in AD^[47]^ and then in amyotrophic lateral sclerosis (ALS) with cognitive impairment (ALSci).^[48,49,50,51,52]^ This modification site can be phosphorylated by multiple kinases related to tauopathy, including GSK3β, JNK, ERK2, and p38,^[53]^ that induce tau aggregation.^[54]^ pThr${}^{175}$ tau induces GSK3β activation and can augment tau phosphorylation at Thr${}^{231}$ and other residues. This results in dissociation of tau from MTs, self-aggregation, and neuronal toxicity.^[55]^

Threonine${}^{231}$ (Thr${}^{231}$) is another important phosphorylation site in tau protein that is phosphorylated or hyperphosphorylated in the cerebrospinal fluid of AD patients, and is correlated with memory loss and progression of AD from mild to severe cognitive impairments.^[56]^

Tau hyperphosphorylation may result from downregulation of phosphatases, especially protein phosphatase 2A by okadaic acid (OA).^[57]^ It was shown that OA treatment induced phosphorylation of tau at Ser${}^{202}$ and Ser${}^{396}$ in cultivated neuroblastoma cells.^[58]^

Cystatin C (CysC) is a cysteine protease inhibitor of cathepsin family, lysosomal proteases, that is widely expressed in various cells and tissues.^[59]^ CysC protein and its gene are upregulated in AD brains, posing a risk factor for late-onset AD.^[60,61]^ CysC does not affect production of cellular amyloid beta peptide (Aβ); however, overexpression of CysC in neurons leads to inhibition of GSK3β turnover, augmentation of GSK3β levels in neurons that promotes GSK3β-tau phosphorylation at Ser${}^{396/404}$, MT instability, and neurodegeneration.^[62,63]^

GSK3β is one of the kinases having an important role in NFT formation and dystrophic neurites by tau phosphorylation in the brain.^[64,65,66,67]^ High activity of GSK3β can be detected in the frontal cortex^[68]^ and hippocampus of AD patients.^[65]^ It is notable that Lithium, as a potent GSK3β inhibitor, is being widely prescribed for neurodegenerative disorders such as AD.

Cyclin-dependent kinase 5 (Cdk5) is a proline-directed Ser/Thre kinase, that can phosphorylate tau at several Ser-Pro and Thre-Pro motifs. Phosphorylation at these residues affects MT stability via dissociation of tau from MTs. Cdk5 is highly expressed in axons and growth cones serving to induce neurite outgrowth and cell migration.^[69]^

Peptidyl-prolyl cis-trans isomerase NIMA-interacting 1 (Pin1), a phospho-Ser/Thr isomerase with a regulatory role on tau function, is known to be a critical factor playing part in the AD development. Pin1 converts cis to trans p-tau whereby preventing pathogenic cis p${}^{231}$-tau accumulation (cistauosis) and maintaining tau in a trans conformation.^[70]^

###  Tau Aggregation

Insoluble tau deposits, resulting from tau misfolding and oligomerization, gradually accumulate in neurons, disrupt cell function, and initiate neurodegeneration.^[71]^ Two hexapeptide repeats, amino acids 306–311 (PHF6, Val-Gln-Ile-Val-Lys-Tyr, VQIVKY) and 317–335 (PHF6*, Val-Gln-Ile-Ile-Lys-Tyr VQIINK), in the C-terminal region of tau protein play a significant role in the formation of β-sheets and fibrillary tangles.^[6,72,73]^ PHF6, located at the beginning of the third MT-binding repeat, is present in all tau isoforms, but PHF6* is located at the beginning of the second MT-binding repeat. It was observed that PHF6 and PHF6* can attach to each other and form tau fibrillary tangles.^[74]^ Tau dimerization can occur by interaction between two PHF6, two PHF6*, or between one PHF6 and one PHF6* motif.^[75]^ Finally, tau oligomers elongate and form cross β-sheet structures, forming the aggregates.^[76]^ Although PHF6 and PHF6* are prone to self-assembly, native tau under physiological conditions is resistant to aggregation. Factors enhancing the self-assembly of tau or neutralizing its charge can induce tau aggregation. Due to the presence of PHF6* encoded by exon 10, 4R tau isoforms are more prone to aggregation than 3R tau isoforms.^[77,78]^ Mutations within the hexapeptide motifs, such as the P301L tau mutation, promote tau aggregation in patients with frontotemporal dementia and parkinsonism linked to chromosome 17 (FTDP-17).^[79]^ In addition to the tendency of exon 10 for aggregation, the N-terminal insert encoded by exon 2 induces tau self-aggregation, whereas the expression of exon 3 has an inhibitory role on aggregation via a process modulated by the expression of exon 10.^[80]^ Also, deletion of the positively charged Lys(K)${}^{280}$ residue can suppress tau self-assembly.^[74,80]^

It was demonstrated that anionic condensing agents can induce tau aggregation. For instance, heparin can bind to the second and third MT-binding repeats, the flanking region, and the N-terminal of tau protein, and induce tau aggregation.^[81,82]^ Fatty acids, polyglutamic acid, and tRNA, can also induce tau aggregation.^[83]^ It has been reported that preventing tau aggregation would rescue neurodegeneration. For example, Curcumin, a tau aggregation inhibitor, efficiently suppresses neurodegeneration in tauopathy mouse models.

###  Pathogenic Role of Tau Protein in Neurodegenerative Diseases

**Figure 1:**
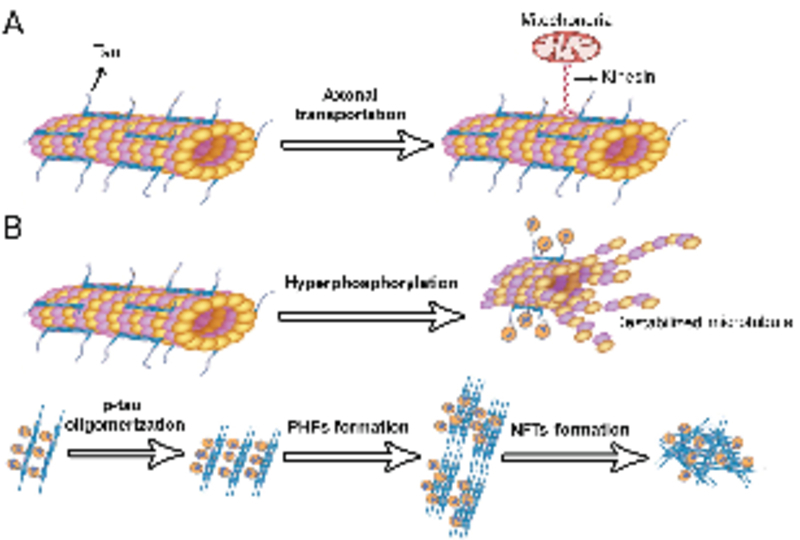
Tau protein. (A) In physiological conditions, tau binds to microtubules by its microtubule binding domain in order to stabilize microtubules for several cellular functions in the cells, such as axonal transportation. (B) In pathological conditions, tau is phosphorylated or hyperphosphorylated (p-tau) by multiple kinases, leading to microtubule destabilization, pairing tau molecules to each other, formation of toxic oligomers, and finally NFT formation.

In contrast to the normal physiological condition in which tau has a stable and unfolded monomeric conformation, in pathological conditions, tau is phosphorylated or hyperphosphorylated and self-aggregates, resulting in pathogenic conformations in neurodegenerative diseases, termed tauopathies.^[7,84]^ The roles of tau in physiological and pathological conditions in the cell are summarized in Figure 1.

Phosphorylation of tau at multiple residues is characteristic of many neurodegenerative diseases, which reduces the affinity of tau to MTs, increases tau self-assembly,^[85,86]^ and finally causes the formation of NFTs.^[87,88]^ The presence of NFTs in specific regions of the brain disrupts synaptic and neuronal communications, leads to progression of memory loss, and produces a rapid impairment of long-term potentiation with induction of toxic functions in the neurons.^[43,89]^

Pathogenic forms of tau may spread in a prion-like manner upon tauopathies, whose molecular mechanisms remain uncertain.^[8,90]^ Cell-to-cell transmission of the tau aggregates causes neuronal cell death and subsequent progression of disease.^[91,92]^ In AD, this process follows a distinct pattern along the neuronal connections from the entorhinal cortex to hippocampal areas and further on through the limbic system.^[93]^ In other tauopathies, this process appears less hierarchical throughout the brain.^[93]^ Moreover, the localization of tau inclusions is widely different in various neurodegenerative diseases.^[42,94,95,96]^

In addition to AD,^[7]^ tau aggregates were detected in a wide range of neurodegenerative diseases including progressive supranuclear palsy (PSP), corticobasal dementia, argyrophilic grain disease, Pick disease, Huntington disease, FTDP-17, ALS, and Parkinson's disease with dementia.^[97,98]^ Unlike AD that has Aβ depositions in addition to tauopathy, PSP is a relatively pure tauopathy in which only tau deposits are seen. PSP was shown to have shared polygenic heritability with Parkinson's disease and ALS, and most of the corresponding genes were clustered around chromosome 17.^[99,100]^ Interestingly, AD, primary age related tauopathy (PART), and aging-related tau astrogliopathy (ARTAG) are the predominant sporadic tauopathies, in which oligodendrocytes serve as targets for seeding and spreading pathologic tau proteins in the white matter.^[101]^ Given that the human retina lacks oligodendrocytes, these cells may not be a component in the pathogenesis of tauopathies in the eye. However, considering the presence of retinal microglial cells and their role in engulfing tau oligomers and induction of inflammation,^[102]^ they may be involved in tau seeding in retinal neurodegenerations.

Tau accumulation is also a result of traumatic brain injury (TBI) and chronic traumatic encephalopathy (CTE), especially in sports that repeatedly expose athletes to mild traumatic brain injury (rmTBI) and in military personnel exposed to repeated traumas.^[103,104,105]^

Mitochondrial dysfunction and reactive oxygen species (ROS) production can induce tau hyperphosphorylation.^[106]^ It has been demonstrated that hyperphosphorylated tau reduces the release of cytochrome C from mitochondria as well as caspase-9 and caspase-3 activity, and protects cells from apoptosis.^[107]^ Moreover, tau via stabilizing β-catenin and increasing its nuclear translocation has the ability to antagonize apoptosis and promote cell survival.^[107,108]^ Tau hyperphosphorylation confers cellular resistance to chemically induced apoptosis through upregulated glycogen synthase kinase-3β (GSK3β) and preserving β-catenin.^[108]^

Tau overexpression causes vascular changes in the cerebral cortex that are accompanied by cortical atrophy. This overexpression in the neurons can lead to dramatic cell-nonautonomous changes in cerebral endothelial cells, alter the integrity of the cerebral microvasculature, and induce neurodegeneration related to vascular abnormalities.^[109]^ A similar situation occurs in AMD, where dramatic neovascular changes accompany neurodegeneration.^[110]^ Additionally, tau overexpression would result in impaired axonal transport, leading to neurodegeneration.

All six tau isoforms are expressed in both soluble and insoluble tau isolates in patients with CTE and CTE-ALS. The expression of oligomerized tau protein and activated GSK3β, pThr${}^{175}$ tau, and pThr${}^{231}$ tau were observed in hippocampal neurons and spinal motor neurons. Phosphorylation of tau at Thr${}^{175}$ and Thr${}^{231}$ and activation of GSK3β are reported features of tauopathy in CTE and CTE-ALS.^[111]^

A direct association has been proposed between Aβ toxicity and tau pathology.^[112,113]^ High levels of Aβ in transgenic mice overexpressing amyloid precursor protein (APP) accelerated tau phosphorylation. Intracerebral injection of Aβ into tau transgenic mice augmented pathogenic conditions.^[114,115]^ Moreover, pathogenic tau augments the secretion of α-synuclein and induces its toxicity by promoting smaller α-synuclein inclusion formation in human neuroglioma cells.^[116]^ It was also shown that α-synuclein can induce tau phosphorylation.^[117,118]^

Cistauosis is a mechanism that occurs before insoluble tau deposition and within days after TBI. Cis p-tau is a pathogenic form of tau protein that is prominently formed in human CTE and uses a prion-like mechanism for its spreading in the brain. Inhibition of cis p-tau by monoclonal antibodies was reported to decrease cellular neurotoxicity, histopathological changes, and behavioral deficits. Cis p-tau is present in cortical axons and cerebrospinal fluid of human TBI and positively correlates with axonal injury. Therefore, antibodies that target this pathogenic form of tau are potential novel treatments for neurodegenerative diseases.^[70]^

###  Tau Isoform Pathology

4R tau isoforms include a fourth MT-binding repeat encoded by exon 10, whereas the 3R tau isoform mRNAs lacks exon 10. The majority of mutations occur in exon 10 or close by. Approximately 40 pathogenic mutations in the *MAPT* gene have been identified, most of them are associated with clinical signs of FTDP-17 with degeneration in the frontotemporal lobar regions.^[119]^ Tau mutations affecting splicing efficacy are located either in exon 10 or intron 10 and result in a change of the 4R/3R tau mRNA ratio.^[120,121]^

All six tau isoforms and tau tangles in the brain of AD patients are formed from both 3R and 4R isoforms with equal ratio, similar to healthy adults;^[122,123,124]^ however, a higher ratio of 4R/3R was observed in PSP in comparison to AD patients and healthy adults.^[124,125]^ Moreover, there have been several cases of PSP and frontotemporal lobar degeneration (FTLD) that had higher 4R tau/3R tau isoforms than FTDP-17 cases.^[124]^

###  Tau Protein in Inflammation

Pathogenic tau molecules, by attaching to each other, form large fibrillar molecules known as tangles. However, there is evidence to suggest that smaller soluble aggregates, named oligomers are the most toxic species and are formed prior to the tangles.^[126,127]^ Tau oligomers may induce inflammatory signaling in the brain of patients with FTLD, AD, and other neurodegenerative diseases. These toxic oligomers are associated with neuroinflammation markers suggesting their role in chronic neuroinflammation, physiological impairments, cellular dysfunction, and ultimately, neurodegeneration.^[128,129,130]^ Oligomers co-localize with astrocytes, microglia, pro-inflammatory cytokines, and high mobility group box-1 protein (HMGB1). Moreover, astrocytes interact with tau oligomers but do not engulf them, while microglia^[130]^ can engulf tau oligomers, secrete them by exosomes, facilitate their propagation, and induce inflammation.^[102]^ Therefore, tau oligomers augment inflammation and cause more damage to cells, which may increase oligomer formation.^[130]^

In addition, in animal models of tauopathy, tau oligomers were detected to be associated with inflammatory cells in the retina, suggesting that the retina can be a valid and non-invasive biomarker for brain degenerative pathologies.^[130]^ It is notable that tau aggregates would induce ROS, resulting in inflammation in the CNS of those tauopathy patients.

###  Pathogenic Role of p-tau in Ocular Neurodegenerative Diseases

The retina is a part of the CNS, containing various cell types, including photoreceptors, horizontal cells, amacrine cells, bipolar cells, and retinal ganglion cells (RGCs), and is easily accessible to noninvasive imaging techniques, such as scanning laser ophthalmoscopy (SLO) and optical coherence tomography (OCT). Identifying retinal pathological changes with these techniques may be used as potential biomarkers for AD,^[131]^ and therefore, ocular screening programs can be planned for identifying tauopathies such as AD.^[132]^ Although the sensitivity and specificity of non-retinal biomarkers has been determined in brain neurodegeneration,^[133]^ there has been no report on the sensitivity and specificity of retinal biomarkers for ocular and brain neurodegenerative disorders. Although there are reports demonstrating tau hyperphosphorylation and aggregation in ocular neurodegeneration, the actual tau pathogenicity upon the disease has not been extensively studied thus far.^[131]^ And so, it seems difficult to clearly demonstrate the superiority of the pathologic form of tau as a marker in conventional behavioral tests and neuroimaging.

Some of the pathological changes in AD patients include reduced thickness of the retinal nerve fiber layer (NFL), attenuation in retinal blood flow and venous diameter, axonal degeneration in optic nerves, decreased number of RGCs, and astrocytosis.^[134,135,136,137]^ Moreover, reduction of macular thickness is inversely associated with the severity of AD.^[138]^ There is a higher incidence of AMD in patients with AD;^[139]^ therefore, there is an strong correlation between AMD and AD incidence and the retinal changes can be used as potential biomarkers for the diagnosis of cerebral neurodegenerative diseases.^[136,140]^ Retinal degeneration in AD was triggered by a pathogenic form of tau via calpain-mediated tau hyperphosphorylation.^[44]^

The presence of tau inclusions in human retinas was discovered for the first time in the corpora amylacea of the optic nerve and the retina.^[141]^ Pathogenic forms of tau in the form of oligomers or aggregations were detected in a variety of ocular disorders such as AMD and glaucoma. Tau was detected in various sublayers of the retina including the inner nuclear layer (INL), inner/outer plexiform layer, and NFL of the retinas from patients with AMD.^[142]^ AMD, one of the leading causes of blindness worldwide,^[143]^ is a complex age-related pathology that occurs as an interplay between oxidative stress, low-grade inflammation, and aberrant accumulations of extracellular protein molecules.^[144]^ Induction of neuroinflammation^[130]^ and aggregation of tau proteins within retinal layers of cases with age-related retinal lesions^[145]^ are two main methods by which tauopathy can act in the pathogenesis of AMD. Additionally, correlations between AD and glaucoma have been well documented.^[146]^ P-tau plays a pathogenic role in the development of glaucomatous optic neuropathy and is upregulated in the retrolaminar region of the optic nerve head in glaucomatous eyes.^[147]^ Moreover, mislocalization of p-tau aggregates was detected in the somatodendritic compartments of RGCs that were subjected to high intraocular pressure.^[147]^ Importantly, tau knock-down using a targeted siRNA protected RGC somas and axons from hypertension-induced damage.^[148]^ Tau oligomers were detected in the retina of an animal model of glaucoma and it was observed that tau inhibition could reduce retinal degeneration.^[148]^ Moreover, it was demonstrated that calpains, proteins belonging to a family of calcium-dependent and non-lysosomal cysteine proteases are activated after ocular hypertension, a condition in which increase of calcium in the retinas and increase of calpain-dependent proteolysis of tau occurs that leads to neuronal cell death.^[149,150]^ It is noteworthy that augmentation of hyperphosphorylated tau has been identified in the vitreous samples of diabetic retinopathies that represent a form of ocular degeneration.^[151]^

AT8 tau with phosphorylation profile at Ser${}^{202}$ and Thr${}^{205}$ has been well characterized, and AT8 antibody was used to stage human neuropathological diseases.^[152]^ AT8 hyperphosphorylated tau was detected in the outer border of the INL and specifically in the inner plexiform layer (IPL) of glaucomatous retinas. In addition, this form of tau colocalizes with parvalbumin in the retinal horizontal cells.^[153]^ AT8 tau protein was also detected with Aβ depositions in various retinal layers including the RGC layer, IPL, INL, outer plexiform layer (OPL), and outer nuclear layer (ONL) in Tg2576 mice.^[154]^

Photoreceptor cells have high energy demands and are significantly affected by ageing.^[155,156]^ Tau aggregates were observed within the cytoplasm of rod and cone photoreceptors, and a positive correlation was observed between age and the number of inclusions in these cells.^[145]^ Conversely, p-tau is specifically accumulated in primate cones while reducing the cellular functions.^[12]^ Diffuse tau aggregates were found in the retinal INL of enucleated eyes that had abnormal retinal changes. They were also detected in the cytoplasm of several photoreceptor cells in patients older than 63 years, and a positive correlation was observed between the patients' age and the numbers of RGCs with tau aggregates. In addition, aggregated tau was found within the cytoplasm of photoreceptor cells in the majority of patients above 63 years of age.^[145]^ OA, one of the positive regulators of p-tau accumulation, was shown to have a destructive role in the cytoskeleton network and growth-cone cells.^[157]^

Transgenic mice expressing P301S mutant human tau model tauopathy develop hyperphosphorylated tau aggregations in the CNS. P301S mutant tau carries a substitution of Ser instead of Pro in exon 10 of the *MAPT* gene.^[158]^ In P301S retinas, hyperphosphorylated tau molecules are aggregated in the nerve fiber and RGC layers. Also, RGC axonal outgrowth did not respond to neurotrophic stimuli in retinal explants cultured from P301S mice, suggesting that pathogenic tau can change neurotrophic signaling.^[159]^ Moreover, the mild tauopathy that developed in RGCs of P301S mice induced functional retinal changes and neuronal dysfunction by disrupting brain-derived neurotrophic factor (BDNF) signaling, via the TrkB receptor that is essential for neuronal survival and synaptic plasticity.^[160]^ Moreover, P301S tau aggregates in RGCs were associated with a reduction in anterograde and retrograde axonal transport in vivo, with a markedly increased effect of excitotoxic injury.^[161]^

Another tau gene mutation is P301L that expresses Leu instead of Pro at position 301 in both the shortest and longest four repeat tau isoforms, and forms NFTs in the CNS.^[162]^ Age-related neurodegenerative changes with significant reduced thickness of retinal INL were observed in P301L mice, and these changes were more pronounced at the peripheral areas and with increasing age. Furthermore, an increase in the size of the RGCs obtained from tau P301L mice was observed with increasing age, in contrast to control mice in which RGC sizes decreased with increasing age.^[163]^

The dynactin complex plays a pivotal role in the transportation network in many cell types by mediating the binding of MT motor complex dynein to its cargoes.^[164]^ Tau and dynactin have extensive interactions, and dynactin attachment to MTs is facilitated by the binding of tau N-terminal domain to the C-terminus of the p150 subunit of dynactin.^[28]^ Mutation in the arginine residue in the N-terminal domain of tau was detected in patients with FTDP-17 that affects tau binding to dynactin, and tau was abnormally distributed in the RGC axons of tau P301S transgenic mice.^[28]^ However, recombinant human tau promoted the attachment of the dynactin complex to axonal microtubules, which indicates a potential role of tau in axonal transportation.^[28]^

Transportation of organelles such as mitochondria and peroxisomes by kinesin molecules was inhibited in rat RGCs with abnormally aggregated tau, evidencing the essential role of tau in kinesin-mediated transportation. Thus, rat RGCs with accumulated tau suffered from loss of energy production and accumulation of ROS due to perturbation of mitochondria and peroxisome function. Moreover, the rate of anterograde transportation that include vesicles necessary for growth cones and synaptic function was slower.^[165]^

Oxidative damage was shown to be associated with death of cones in retinitis pigmentosa, and of both photoreceptor cell types, in AMD.^[166]^ Thioredoxin 1 (TRX1) in the retina plays a protective role against photooxidative damage.^[167]^ Rod-derived cone viability factor (RdCVF) is a member of the thioredoxins family^[168]^ and a trophic factor secreted by rods for maintaining cone viability and functionality.^[169]^ In murine models, RdCVF is encoded by the *Nxnl1 *gene that also encodes for a second polypeptide, RdCVFL, by alternative splicing. RdCVFL inhibits tau phosphorylation and protects tau from oxidative damage.^[170]^ Upregulation of tau phosphorylation has been well demonstrated in the retinas of *Nxnl1*-/- mice.^[171,172]^ Therefore, therapeutic modalities augmenting the *NXNL1 *gene or the corresponding protein may be promising for the treatment of cerebral and ocular neurodegeneration.

###  Therapeutic Strategies for Targeting Pathologic Tau 

The pathogenic tau molecule is an excellent therapeutic target for neurodegenerative diseases using several strategies such as reducing levels, altering post-translational modifications, or blocking propagation of pathogenic tau.

Several tauopathy-based targeted therapies for neurodegenerative diseases have been introduced, such as suppressing tau misfolding,^[173]^ targeting tau acetylation,^[174]^ inhibiting tau-induced proteasome impairment,^[175]^ and tau immunotherapy.^[176,177,178,179,180]^ Several therapeutic modalities for inhibiting tau aggregation have been reported,^[19,181]^ among which, the use of small molecules has recently gained much interest.

Several small molecules exhibit anti-tau aggregation properties;^[182,183]^ one of these is D-enantiomeric peptides that has modulating mechanisms on tau self-aggregation.^[184]^ Other small molecules that were shown to have anti-tau aggregation effects have an eight amino acid peptide named NAP (Davunetide).^[185]^ Clinically, NAP (davunetide) exhibited its efficacy in prodromal AD patients that had equal 3R and 4R Tau isoforms but not in the PSP cases that had increased 4R Tau.^[186]^ Cationic small molecules and cationic osmolyte urea also have inhibitory effects on tau accumulation.^[187,188,189]^ Moreover, the inhibitory effect of a cationic polymer polyethyleneimine and a cationic polypeptide arginine on the aggregation of VQIVYK and GKVQIINKLDL peptides in tau protein was also observed.^[190]^

Antisense oligonucleotides are other small molecules that selectively downregulate human tau at mRNA and protein levels in adult mouse brain that express mutant P301S human tau.^[191]^ Moreover, the chaperone Artemin was found to be an effective inhibitor of tau inclusions in both physiologic and supraphysiologic concentrations, in a dose-dependent manner and in a cell-free model system. This supports the idea that Artemin can be a therapeutic option for people with AD.^[192]^

Although several specific small molecules for targeting pathogenic tau have been discovered, more studies are needed to illustrate the safety and efficacy of these molecules as novel drugs. More importantly, it is of crucial importance to clarify which epitope to target?

We herein studied latest reports on tau pathology; especially in ocular neurodegeneration. Although we may not clearly describe the pathogenic tau species playing part in ocular neurodegeneration, however, due to similarities between brain and eye neurodegeneration, the aforementioned treatment modalities for brain tauopathies sound like efficient therapeutic strategies in fighting with the ocular neurodegeneration.

##  Summary

The current review, based on numerous studies on the role of pathogenic tau in neurodegeneration, attempts to highlight the connection between brain and eye diseases. Pathogenic tau protein exerts destructive effects that are associated with cerebral degenerations such as AD, PSP, and TBI, and ocular neurodegenerative disorders such as AMD, glaucoma/ocular hypertension, and probably diabetic retinopathy. The destructive effects of the pathogenic form of tau can be exerted by several forms of tau such as soluble oligomers, insoluble aggregates, or even by cis p-tau which is an early driver of pathogenic tau. Considering this evidence, targeted therapy based on pathogenic tau could be a promising therapeutic for patients with CNS or ocular neurodegeneration, to not only prevent the more destructive effects of pathogenic tau, but also restore normal functioning to neural cells. Moreover, with the identification of tauopathy-related retinal changes with noninvasive imaging techniques, screening programs for the early diagnosis of CNS tauopathies such as AD can be planned.

##  Financial Support and Sponsorship

Nil.

##  Conflicts of Interest

There are no conflicts of interest.
